# A mixed-methods investigation of incident Hemodialysis access in a safety-net population

**DOI:** 10.1186/s12882-017-0700-9

**Published:** 2017-09-02

**Authors:** Nicole C. Rich, Shant M. Vartanian, Shimi Sharief, Daniel J. Freitas, Delphine S. Tuot

**Affiliations:** 1 Department of Surgery, General Surgery Residency Program, U.C. San Francisco, 513 Parnassus Ave., S321, San Francisco, CA 94143-0470 USA; 2 Department of Surgery, Division of Vascular & Endovascular Surgery, U.C. San Francisco, 400 Parnassus Ave., Suite A-501, San Francisco, CA 94143 USA; 3 Department of Medicine, Division of Nephrology, U.C. San Francisco, 505 Parnassus Ave, San Francisco, CA 94143 USA; 4 School of Medicine, U.C. San Francisco, 505 Parnassus Ave, San Francisco, CA 94143 USA

**Keywords:** Hemodialysis access, Vascular access, Arteriovenous fistula, Vascular surgery, Disparities, Safety-net

## Abstract

**Background:**

Despite improved health outcomes associated with arteriovenous fistulas, 80% of Americans initiate hemodialysis using a catheter, influenced by low socioeconomic status among other factors. Risk factors for incident catheter use in safety-net populations are unknown. Our objective was to identify factors associated with incident catheter use among hemodialysis patients at one safety-net hospital, with a goal of informing fistula placement initiatives targeted at safety-net populations more generally.

**Methods:**

We performed a retrospective review of all incident hemodialysis patients at a single urban safety-net hospital from January 1, 2010 - December 31, 2015 (*n* = 241), as well as semi-structured interviews with a multi-lingual convenience sample of patients (*n* = 10) from this cohort. The primary outcome was incident vascular access modality. Multivariable logistic regression was used to identify factors associated with incident catheter use. Interview transcripts were coded using a directed content analysis framework based on a model describing barriers to healthcare access.

**Results:**

Subjects were 61.8% male, racially/ethnically diverse (19.5% white, 29.5% black, 28.6% Hispanic, 17.4% Asian), with a mean age of 52.4 years. Eighty-eight percent initiated hemodialysis using a catheter. In multivariable analysis, longer duration of nephrology care was associated with decreased catheter use (>12 months vs. 0–6 months: adjusted Odds Ratio [aOR] 0.07, 95% CI 0.02–0.23, *p* < 0.001), whereas uninsured status increased odds of catheter use (aOR 3.96, 1.23–12.76, *p* = 0.02). There was a decrease in catheter use after vascular surgery services became available in-hospital (OR 0.40, 95% CI 0.16–0.98, *p* = 0.04), however this association was not significant in multivariable analysis (aOR 0.48, 0.17–1.36, *p* = 0.17). During interviews, patients cited emotional responses to disease, lack of social and financial resources, and limited health knowledge as barriers to obtaining fistula surgery.

**Conclusions:**

The rate of catheter use in this urban safety-net population is above the national average. Access to health insurance, early referrals to nephrology, and provision of in-hospital vascular surgery should be prioritized in the safety-net. Additionally, services that support patients’ emotional and learning needs may decrease delays in fistula placement.

## Background

The type of vascular access used for incident hemodialysis (HD) has a strong correlation with patient outcomes and resource utilization. Arteriovenous fistulas (AVFs) are associated with decreased morbidity, mortality, and healthcare costs when compared with central venous catheters (CVCs) [1–5]. Of the approximately 117,000 patients with end stage renal disease (ESRD) who initiated HD in the U.S. in 2013, only 17.1% started with an AVF, while 80.2% used a CVC [3, 6]. This pattern of incident HD access has changed very little over the past ten years, despite consensus recommendations from vascular surgeons, nephrologists, and the U.S. government dating back to 1997 that suggest we should aim for a rate of at least 50% AVF use at HD initiation [6–8].

National data reveal substantial variation in incident AVF rates from 11.1% to 22.2% among regional ESRD networks, and significant disparities in incident AVF use by race, gender, and insurance status [9, 10]. Because blacks, Hispanics, women, and the uninsured are significantly less likely to have a functioning AVF at the time of HD initiation, these vulnerable populations disproportionately bear the burden of complications due to CVC use. This indicates a need to better understand the challenges that vulnerable populations face in attaining vascular access in a timely fashion, as they may not mirror national trends. [6, 11]

The aim of this study was to identify and describe factors associated with incident CVC use among a diverse, low-income, multi-lingual HD population at one urban safety-net hospital, with a goal of informing regional and national AVF placement initiatives pertinent to safety-net populations. We hypothesized that an urban, uniformly low-income, and racially diverse population of patients with ESRD may face different barriers to AVF placement than those that are pertinent at the national level.

## Methods

### Study design

This study was conducted in an outpatient HD unit that is affiliated with an urban safety-net hospital. We performed a retrospective review of all adults who initiated HD at this hospital either as inpatients or outpatients, and who went on to continue outpatient HD treatments at this facility, with HD initiation dates between January 1st, 2010 and December 31st, 2015. Patients were excluded if they were initiated on HD briefly while being bridged to peritoneal dialysis (*n* = 11). Data were collected primarily from the Centers for Medicare & Medicaid Services Form 2728, which is submitted for each patient at the time of HD initiation. The primary outcome was vascular access modality at the time of HD initiation, modeled as a binary variable: surgical access (which included both AVF and arteriovenous [AV] graft) or CVC (with or without maturing surgical access in place). Predictors included variables that have been described nationally, but also those that might disproportionately affect our population: age, gender, race/ethnicity (white, black, Hispanic, Asian, other/unknown), limited English proficiency (obtained from electronic medical records), comorbid conditions previously identified as significantly correlated with patient survival in the ESRD population (congestive heart failure, hypertension, chronic obstructive pulmonary disease (COPD), peripheral vascular disease, amputation, diabetes, drug abuse, cancer), body mass index (BMI), etiology of renal failure, income quintile (estimated using census data for patient zip code), insurance status (Medicare only, Medicaid only, Medicare & Medicaid, none, other), duration of pre-hemodialysis nephrology care, and year of HD initiation. [12–14] Because dedicated vascular surgical services became available to this population in August 2012, we constructed a pre/post 2012 term to examine whether access to on-site vascular surgery services was associated with incident CVC use.

### Statistical analysis

Using Fisher’s exact test, we looked for univariate associations with incident CVC use among predictor variables. We then constructed a restricted multivariable logistic model to predict odds of CVC use, including variables that were significant in the univariate analysis at a *p*-value of <0.05, or that provided demographic information necessary to maintain generalizability. All statistical analyses were performed using STATA software (version 13.1; Stata-Corp LP).

### Qualitative methods

In order to contextualize our quantitative results and develop strategies to address high rates of incident CVC use, we performed one-on-one interviews with a sample of patients from the study population who had initiated HD in the prior year. Primary nephrologists were asked to exclude potential interview patients with psychiatric co-morbidities and dementia. Patients deemed appropriate were subsequently approached and asked to provide informed consent. A single investigator (NR) performed all interviews using a standard list of questions regarding the barriers patients had experienced in the process of obtaining vascular access for HD, and their suggestions to improve the process. Interviews with patients with limited English proficiency were conducted with the aid of professional interpreter services via telephone. All interviews were audiotaped and professionally transcribed. Interview transcripts were interpreted by using directed content analysis, in which a previously described theoretical model is applied and extended in order to create initial coding categories [15]. A theoretical model describing multilevel barriers leading to healthcare disparities was extended to incorporate patient experiences and suggestions for improving care [16]. Three broad coding categories were created: barriers to care, attitudes toward care, and suggestions for care. Three investigators independently read and coded interview transcripts using this framework and then arrived at a consensus on the main themes encountered within each broad coding category. Based on agreement upon the adequate detail and variety of data within each theme, the authors agreed that saturation had been reached [17]. The University of California, San Francisco institutional review board approved all portions of this study.

## Results

### Patient characteristics

Our cohort consisted of 241 patients with a mean age of 52.4 years (range = 21–86), of whom 61.8% were male. They were racially and ethnically diverse (19.5% white, 29.5% black, 28.6% Hispanic, 17.4% Asian). Forty-two percent were uninsured and an additional 39% were covered only by Medicaid (Table 1). Twenty-four percent had limited English proficiency (LEP), speaking a variety of primary languages including Spanish, Chinese, Vietnamese, and Tagalog.

**Table 1:** Demographics of Retrospective Cohort by type of Incident Vascular Access

	Total *n* = 241	AVF/AV Graft *n* = 29 (12.1%)	Central Venous Catheter (CVC) *n* = 212 (87.9%)	*p* value for association with access type^a^
Age, mean (SD)	52.4 (12.2)	52.6 (11.6)	52.4 (12.4)	0.94
Female sex, %	38.2	37.9	38.2	>0.99
Race/Ethnicity, %				0.83
white	19.5	13.8	20.3	
black	29.5	27.6	29.7	
Hispanic	28.6	31.0	28.3	
Asian	17.4	24.1	16.5	
other/unknown	5.0	3.4	5.2	
Limited English proficiency, %	24.1	31.0	23.0	0.36
Comorbidity prevalence, %				
congestive heart failure	20.8	20.7	20.8	>0.99
hypertension	86.7	93.1	85.8	0.39
COPD^b^	6.2	3.4	6.6	>0.99
peripheral vascular disease	3.3	6.9	2.8	0.25
amputation	1.7	3.4	1.4	0.40
diabetes mellitus	44.4	31.0	46.2	0.16
drug abuse	28.2	24.1	28.8	0.67
cancer	2.5	3.4	2.4	0.54
Body mass index, mean (SD)	27.5 (7.9)	29.2 (9.6)	27.2 (7.7)	0.20
Etiology of renal failure, %				0.48
diabetes mellitus	34.4	31.0	34.9	
hypertension	12.5	17.2	11.8	
glomerulonephritis	17.0	24.1	16.0	
other	36.1	27.6	37.3	
Income quintile, mean (SD)	2.27 (1.2)	2.24 (1.1)	2.28 (1.2)	0.87
Insurance status, %				0.02*
Medicaid	38.6	55.2	36.3	
Medicare	9.5	10.3	9.4	
Medicare and Medi-Cal	2.9	6.9	2.4	
other	7.1	10.3	6.6	
none	41.9	17.2	45.2	
Duration of nephrology care, %				<0.001*
none - 6 months	60.6	20.7	66.0	
6–12 months	17.0	20.7	16.5	
> 12 months	22.4	58.6	17.6	
Period of HD initiation^c^, %				0.045*
Pre August, 2012	41.9	6.9	93.1	
Post August, 2012	58.1	15.7	84.3	

^a^ *p* values obtained using Fisher’s exact test for categorical variables and t-test for continuous variables

^b^COPD = chronic obstructive pulmonary disease

^c^Dedicated vascular surgical services became available at ZSFG during August 2012

*significant at *p* < 0.05

**Fig. 1:**
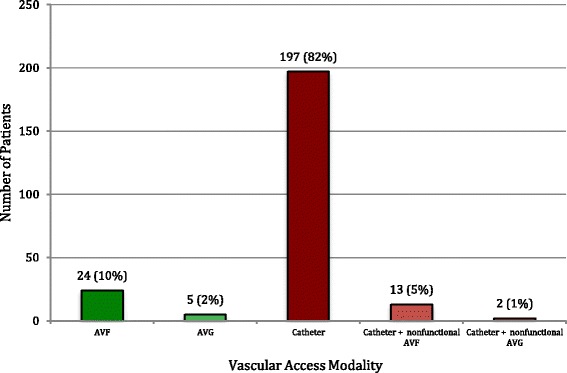
Incident Vascular Access Modality 2010–2015

**Fig. 2:**
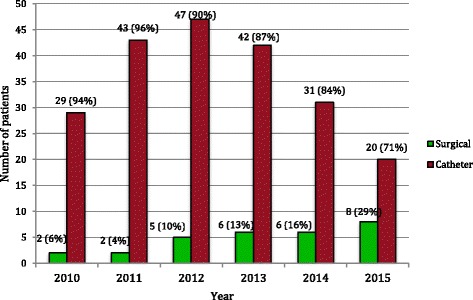
Incident Vascular Access Modality by Year

Overall, 10.0% of patients initiated HD using an AVF and 2.1% using an AV graft, while 87.9% of patients initiated HD using a CVC, which included 6.2% who had a maturing AVF or nonfunctional AV graft in place (Fig. 1). When examined by year of HD initiation, an increase in the annual rate of incident surgical access (AVF or AV graft) was identified (Fig. 2).

### Associations with incident CVC use

Odds of initiating HD with a CVC, as predicted by univariate logistic regression and a restricted multivariable logistic model are displayed in Table 2. Patient clinical and demographic characteristics were not significantly associated with incident CVC use. Uninsured status, duration of pre-dialysis nephrology care, and HD initiation pre/post 2012 were significantly associated with incident CVC use in univariate analyses. Of these predictors, all remained significant predictors of incident CVC use in the multivariable logistic model except for timing of HD initiation (odds ratio in univariate analysis =0.40, 95% CI 0.16–0.98; adjusted odds ratio [aOR] in multivariable analysis =0.48, 0.17–1.36).

**Table 2:** Associations of Predictor Variables with Incident CVC use

	Univariate logistic regression	Multivariable logistic regression (restricted model)
	OR^a^ (95% CI^b^)	aOR^c^ (95% CI)
Age	1.00 (0.97–1.03)	1.03 (0.99–1.07)
Female sex	1.01 (0.45–2.25)	0.90 (0.36–2.28)
Race/Ethnicity		
white	ref	ref
black	0.73 (0.21–2.59)	2.16 (0.46–10.26)
Hispanic	0.62 (0.18–2.15)	1.90 (0.42–8.61)
Asian	0.47 (0.13–1.72)	1.08 (0.22–5.22)
other/unknown	1.02 (0.10–10.10)	10.55 (0.80–140.04)
Limited English Proficiency	0.67 (0.29–1.56)	-
Comorbidity		
congestive heart failure	1.00 (0.39–2.62)	-
hypertension	0.45 (0.10–1.99)	-
COPD^d^	1.98 (0.25–15.64)	-
peripheral vascular disease	0.39 (0.08–2.05)	-
amputation	0.40 (0.04–4.00)	-
diabetes mellitus	1.91 (0.83–4.39)	-
drug abuse	1.27 (0.52–3.13)	-
cancer	0.68 (0.08–6.00)	-
Body mass index	0.97 (0.93–1.02)	-
Etiology of renal failure		
diabetes mellitus	ref	-
hypertension	0.61 (0.19–1.99)	-
glomerulonephritis	0.59 (0.20–1.72)	-
other	1.20 (0.44–3.28)	-
Income quintile	1.03 (0.73–1.44)	-
Insurance status		
Medicaid	ref	ref
Medicare	1.39 (0.37–5.23)	1.53 (0.31–7.45)
Medicare and Medi-Cal	0.52 (0.09–2.92)	0.20 (0.03–1.48)
other	0.97 (0.25–3.77)	1.77 (0.36–8.76)
none	3.99 (1.40–11.38)*	3.96 (1.23–12.76)*
Duration of nephrology care		
none - 6 months	ref	ref
6–12 months	0.25 (0.08–0.82)*	0.23 (0.06–0.90)*
> 12 months	0.09 (0.03–0.25)*	0.07 (0.02–0.23)*
HD initiation post 2012	0.40 (0.16–0.98)*	0.48 (0.17–1.36)

^a^OR = odds ratio

^b^CI = confidence interval

^c^aOR = adjusted odds ratio

^d^COPD = chronic obstructive pulmonary disease

*significant at *p* < 0.05

In the multivariable model, patients who were uninsured had a nearly four-fold increase in odds of incident CVC use compared to patients with Medicaid (aOR = 3.96, 1.23–12.76). We found a graded association between duration of pre-dialysis nephrology care and odds of incident CVC use. Compared to patients with less than six months of pre-dialysis nephrology care, those with 6–12 months had a 77% decreased odds (aOR = 0.23, 0.06–0.90), and those with over 12 months had a 93% decreased odds of initiating HD with a CVC (aOR = 0.07, 0.02–0.23).

### Themes from patient interviews

Ten patients who had initiated HD within the prior year participated in the interviews. Half of the participants were English speakers and the remainder spoke a variety of other languages. Eight of the participants were male, with a mean age of 54.4 years (range = 39–65). Half of the participants had initiated HD using an AVF and half had initiated with a CVC. The major themes in patient responses are presented in Table 3, categorized into three broad groups: stress & emotional barriers, limited resources, and patient suggestions for vascular access care coordination.

**Table 3:** Themes in Patient Responses Regarding AVF Placement

Stress & Emotional Barriers	Limited Resources	Patient Suggestions for Care
Emotional responses to surgery: fear, denial, depression, frustration	Lack of information, misinformation regarding disease	Address emotional needs of pre dialysis patients
Anxiety regarding pain of surgery / fistula cannulation	Lack of health insurance coverage or primary care	Individualize delivery of information for each patient’s learning style
Difficulty engaging in care and processing information due to depression / anxiety	Missed appointments due to lack of transportation or illness	Provide opportunities to interact with experienced HD patients
Lack of control and uncertainty	Limited English proficiency	Provide clear explanation of what fistula entails and potential complications
Doctor patient trust	Housing instability, drug use, and poverty in living environment	Improve engagement with own medical care
Concerns regarding appearance of fistula	Late diagnosis / urgent hemodialysis start	Build trust / relationship with doctor

### Stress and emotional barriers

Participants experienced strong emotional responses to either their diagnosis of renal failure, or the impending need for HD and/or surgery. They reported feeling anxiety, depression, panic, denial, and frustration. Some participants described how these emotional states impaired their ability to make the decisions necessary to coordinate surgery for AVF placement. One patient who started HD using an AVF had the insight to look back at his decision making in the months leading to HD initiation and state:

“I think a patient can be traumatized by the experience of now knowing that the patient needs dialysis and that sometimes can create anxiety and the slowing of the thinking process and making prudent decisions, regardless of the level of responsibility that that patient has exhibited in the past.”.

Attitudes toward healthcare providers at the dialysis unit were overwhelmingly positive. Despite this, some patients reported that their emotional responses made it difficult to participate in their own care. Multiple participants expressed a deep sense of loss at the time of HD initiation that resulted from no longer being able to work or travel, newly imposed dietary restrictions, and the physical changes that their disease or even the AVF surgery itself caused. Some participants were self-conscious about the way the AVF looked, commenting on new considerations for how their clothing concealed the AVF. One participant who initiated HD using an AVF described all of this as “an abnormal human situation.”

### Limited resources

Misconceptions and poor quality information were barriers to timely AVF placement. Multiple participants reported anticipating substantial pain from AVF surgery and cannulation, only to discover that their fears overshadowed the reality. One participant who started HD using a CVC wished he had “somebody to lay it out and give you an idea of what a fistula is all about. That would’ve helped tremendously. . . all of my information came in pieces and bits.” Other participants reported heightened anxiety caused by misinformation that they received from acquaintances. One participant who started HD using an AVF stated, “I just get a rundown in the streets, people dying, they take dope on dialysis and die.”.

Limited social and financial resources associated with homelessness and poverty were barriers to complying with the care needed to have an AVF placed. One participant who attributed his kidney disease to years of drug abuse and had started HD using a CVC stated, “I’m still struggling with the things that got me to this point right here. The environment I'm in and the area I stay at, it’s just—it’s bad.” Other participants who had initiated HD using a CVC also cited a lack of reliable transportation, and poor social support during times of illness as barriers to keeping medical appointments before initiating HD.

Participants’ responses regarding language as a potential barrier to care were mixed. Most reported that they were able to easily get interpreter services when needed. However, one participant with LEP who had initiated HD using a CVC did describe challenges in communicating with her doctor, stating, “Who do I talk to? Everyone here only speaks English.” Additionally, participants with LEP who had initiated HD using a CVC identified a lack of insurance coverage as a barrier to receiving pre-ESRD care, whereas English speakers did not. LEP participants described inconsistent primary care, limited access to essential medications, and deficiencies in their own medical knowledge that contributed to progression of kidney disease, delays in ESRD diagnosis, and delayed AVF placement.

### Patient suggestions for care

Enhanced delivery of information was a common desire when participants were asked for suggestions to improve timely AVF placement. Participants reported that more information would have helped to allay their fears and shape their expectations for the better. Opinions on the best way to receive information varied widely; suggestions included educational videos, face-to-face interactions, printed material, or online forums. One participant who started HD using an AVF stated, “I think that new patients can benefit from seeing what current patients experience. . . as opposed to just watching a[n educational] video.” This participant felt that an experienced patient could describe “the physical effects and the psychological effects” of AVF placement and HD more effectively than a doctor could. Another participant who had started HD using an AVF cited an interaction with another patient that helped him overcome his fear of AVF cannulation: “He told me, he says he was scared the first time he did it. But he said once you did it, no problem.”.

Multiple participants who had initiated HD using both AVFs and CVCs suggested that a group setting would be ideal for pre-dialysis patients to meet with more experienced patients who could address their questions and anxieties. One participant proposed: “Why don’t they have like an AA meeting and have a bunch of people. . . share about dialysis and stuff.” Another participant suggested that patients gather to “drink coffee or something and talk about their experience. Not a big group, just a few people. ‘How are you feeling? Are you okay?’”.

## Discussion

In our cohort of incident HD patients from a diverse, urban safety-net population, the rate of incident CVC use was 87.9%, compared to a national average of 80.2% [9, 18]. Factors that were significantly correlated with incident CVC use in this population were those describing access to health care services, including a lack of health insurance, shorter duration of nephrology care, and initiation of HD during the period when in-hospital vascular surgical services were unavailable. Our qualitative results suggested that addressing the emotional needs and individual learning styles of patients who are new to HD, using methods including peer support, would facilitate earlier AVF placement.

Multiple prior studies have identified longer duration of pre-dialysis nephrology care as the strongest predictor of decreasing incident CVC use. [2, 10, 19–23] This graded association was significant in our cohort as well, supporting an assertion that earlier nephrology referrals are key to increasing rates of incident AVF usage, regardless of the population. In addition, we observed a strong association between uninsured status and higher incident CVC use in our cohort. This suggests that efforts to increase insurance coverage may be a critical component of targeted initiatives to decrease incident CVC use in safety-net populations.

The association we observed between in-hospital vascular surgical services and lower incident CVC use among ESRD patients has not been previously reported. Although this association did not reach significance in the multivariable model, this may have been due to the small size of our cohort. Despite this limitation, our data suggest that the provision of dedicated vascular surgery services in the public hospital deserves further attention and study as a means to decrease incident CVC use in safety-net populations.

In distinction to national data, in our cohort we did not observe racial and gender disparities in incident CVC use. Additionally, we did not see significant variation in incident access modality by English proficiency or socioeconomic status. These results are consistent with the safety-net setting, where diversity and low socioeconomic status are the norm rather than factors which serve to stratify patients.

Our qualitative results are consistent with literature documenting a high prevalence of depression and associated difficulty with medical decision-making among patients with chronic kidney disease [24, 25]. Our study participants expressed interest in interacting with more experienced patients, and a desire for more information to aid in their decision-making and to address their anxiety regarding AVF surgery. Patient preference for educational modality varied widely, indicating that a multi-modality approach may be the best suited to meet patients’ varied learning styles. Prior studies have shown that peer support and multi-modality patient education are effective methods of addressing ESRD patients’ concerns, supporting them emotionally, and facilitating their ability to make necessary medical decisions [25, 26]. These types of resources may be especially pertinent for patients in the safety-net, who often have limited psychosocial support mechanisms and low health literacy.

The limitations of our study include the retrospective nature of the cohort, which precludes determination of causality. Additionally, variability in the quality and completeness of the data recorded on form 2728 may have introduced bias into our results. Individual level variables, including educational level and household income, that are not captured on form 2728 or in our electronic medical record would have been of interest as predictors and were not available for our analysis. Finally, the small sample size of both the retrospective cohort and the qualitative study limit the power of our analysis and its generalizability to other safety-net populations, which cannot be assumed to share the same clinical and demographic features.

## Conclusions

Despite the study’s limitations, it provides important insight into the vascular surgical care of a safety-net ESRD population. This is an important first step towards characterizing CVC use in the safety-net, so that this “hot spot” for CVC use and its attendant complications can be addressed using the most effective approaches. Our results suggest that increasing rates of health insurance, promoting early nephrology referrals, prioritizing the availability of in-hospital surgical subspecialty services in the safety-net, and offering ESRD patients peer support and multi-modality education may be the most effective ways to decrease incident CVC use in safety-net populations.

## Abbreviations

aOR: Adjusted odds ratio
AV: Arteriovenous
AVF: Arteriovenous fistula
BMI: Body mass index
COPD: Chronic obstructive pulmonary disease
CVC: Central venous catheter
ESRD: End stage renal disease
HD: Hemodialysis
LEP: Limited English proficiency

## References

[1] Ravani P, Palmer SC, Oliver MJ (2013). Associations between Hemodialysis access type and clinical outcomes: a systematic review. J Am Soc Nephrol.

[2] Zarkowsky DS, Arhuidese IJ, Hicks CW (2015). Racial/ethnic disparities associated with initial Hemodialysis access. JAMA Surg..

[3] Malas MB, Canner JK, Hicks CW (2015). Trends in incident Hemodialysis access and mortality. JAMA Surg..

[4] Allon M, Dinwiddie L, Lacson E (2011). Medicare reimbursement policies and Hemodialysis vascular access outcomes: a need for change. J Am Soc Nephrol.

[5] Solid CA, Carlin C (2012). Timing of Arteriovenous fistula placement and Medicare costs during dialysis initiation. Am J Nephrol.

[6] Hall YN (2012). Racial and ethnic disparities in end stage renal disease: access failure. Clin J Am Soc Nephrol.

[7] Weinhandl E, Constantini E, Everson S, et al. Peer Kidney Care Initiative 2014 Report: Dialysis Care and Outcomes in the United States. Am J Kidney Dis. 2015;65(6) (suppl1):S1–S140. 10.1053/j.ajkd.2015.03.021 26003780

[8] ESRD National Coordinating Center. Fistula First Catheter Last. http://www.esrdncc.org/en/fistula-first-catheter-last. Accessed 9 May 2016.

[9] USRDS. USRDS Annual Data Report 2015, Volume 2, Chapter 1. usrds.org. https://www.usrds.org/2015/view/v2_01.aspx. Accessed May 9, 2016.

[10] Zarkowsky DS, Hicks CW, Arhuidese I (2015). Quality improvement targets for regional variation in surgical end-stage renal disease care. JAMA Surg.

[11] Nee R, Yuan CM, Hurst FP, Jindal RM, Agodoa LY, Abbott KC. Impact of poverty and race on pre-end-stage renal disease care among dialysis patients in the United States. Clin Kidney J. 10(1):55–61. doi:10.1093/ckj/sfw098. 10.1093/ckj/sfw098 PMC5469551 28638604

[12] Environmental Systems Research Institute. ziptapestry. esri.com/data/esri_data/ziptapestry. http://www.esri.com/data/esri_data/ziptapestry. Accessed Apr 15, 2016.

[13] Miskulin D, Bragg-Gresham J, Gillespie BW (2009). Key Comorbid conditions that are predictive of survival among Hemodialysis patients. Clin J Am Soc Nephrol.

[14] Nee R, Moon DS, Jindal RM (2015). Impact of poverty and health care insurance on Arteriovenous fistula use among incident Hemodialysis patients. Am J Nephrol.

[15] Hsieh HF (2005). Three approaches to qualitative content analysis. Qual Health Res.

[16] Powe NR (2008). Let's get serious about racial and ethnic disparities. J Am Soc Nephrol.

[17] Fusch PI, Ness LR (2015). Are we there yet? Data saturation in qualitative research. Qual Rep.

[18] USRDS. USRDS Annual Data Report 2015, Volume 2, Chapter 4. usrds.org. https://www.usrds.org/2015/view/v2_04.aspx. Accessed May 9, 2016.

[19] Wasse H, Hopson SD, McClellan W (2010). Racial and gender differences in Arteriovenous fistula use among incident Hemodialysis patients. Am J Nephrol.

[20] Arce CM, Mitani AA, Goldstein BA, Winkelmayer WC (2012). Hispanic ethnicity and vascular access use in patients initiating Hemodialysis in the United States. Clin J Am Soc Nephrol.

[21] Patibandla BK, Narra A, DeSilva R (2013). Disparities in arteriovenous fistula placement in older hemodialysis patients. Hemodial Int.

[22] Goldfarb-Rumyantzev AS, Syed W, Patibandla BK (2014). Geographic disparities in arteriovenous fistula placement in patients approaching hemodialysis in the United States. Hemodial Int.

[23] Hurst FP, Abbott KC, Raj D (2010). Arteriovenous fistulas among incident Hemodialysis patients in department of defense and veterans affairs facilities. J Am Soc Nephrol.

[24] Palmer S, Vecchio M, Craig JC (2013). Prevalence of depression in chronic kidney disease: systematic review and meta-analysis of observational studies. Kidney Int.

[25] Green JA, Boulware LE (2016). Patient education and support during CKD transitions: when the possible becomes probable. Adv Chronic Kidney Dis.

[26] Hughes J, Wood E, Smith G (2009). Exploring kidney patients’ experiences of receiving individual peer support. Health Expect.

